# Phubbing in Students: New Evidence for a Spanish Short Form of the Phubbing Scale (PS-6)

**DOI:** 10.3390/healthcare12232459

**Published:** 2024-12-05

**Authors:** Javier Ortuño-Sierra, Fermín Navaridas-Nalda, Oliver Mason, Ana Ciarreta-López, Noelia Barbed-Castrejón

**Affiliations:** 1 Educational Sciences Department, University of La Rioja, 26004 Logroño, Spain; javier.ortuno@unirioja.es (J.O.-S.); fermin.navaridas@unirioja.es (F.N.-N.); ana.ciarreta@unirioja.es (A.C.-L.); 2 Psychology Department, Surrey University, Guildford GU2 7XH, UK; o.mason@surrey.ac.uk

**Keywords:** phubbing, phubbing scale, adolescents, students, mental health

## Abstract

Background: Phubbing—the act of snubbing someone by concentrating on a smartphone—is claimed to be an increasing, even normative, behavior receiving more research attention. However, evidence about the adequacy of instruments that allow screening for phubbing is limited. Objectives: Thus, the main purpose of the present study was to analyze the psychometric properties of the Phubbing scale in a sample of adolescents and young adults and present a reliable and valid short form. Methods: The final sample included a total of 1351 adolescent students (54.25% males), aged between 12 and 21. The study used a self-administered questionnaire with different scales for collecting data. Results: A two-dimensional model of a short version of the PS with 6 items (PS-6) displayed appropriate goodness-of-fit indices. Strong measurement invariance for the two-dimensional model across gender and educational levels was confirmed. McDonald’s Omega coefficients for the Communication, the Obsession, and the Total score of the PS-6 were 0.617, 0.669, and 0.701, respectively. The PS-6 was positively associated with several indicators of psychological difficulties and negatively associated with psychological well-being and self-esteem. Conclusions: The present study contributes valuable information about the psychometric adequacy of the PS-6, a short instrument that allows screening for Phubbing in adolescents and young adults.

## 1. Introduction

The term ‘phubbing’ combines ‘phone’ and ‘snubbing’, defining the behavior of prioritizing smartphone engagement over interpersonal interactions [1,2,3].

This phenomenon may be especially problematic during adolescence and young adulthood, life stages that are formative for social interaction, mental health, and well-being [4]. Various studies have established connections between phubbing and mental health issues, poorer communication quality [5,6], diminished interpersonal interactions [7,8], and tendencies towards addictive behaviors [9]. Worth noting, previous research revealed a strong connection between internet addiction, phone use, and aggressive behavior during adolescence. This stage of life is relevant for establishing social relationships, and the misuse of the phone and internet could lead to different emotional and behavioral problems [10]. Furthermore, these connections can be used to explain phubbing as a multidimensional construct that can be explained from multiple perspectives. With this regard, some studies indicated the possible relationship between phubbing and personality traits like Machiavellianism, narcissism, and psychopathy (also known as the Dark Triad) [11]. Nonetheless, only vulnerable narcissism has been found to be related to phubbing [11]. Recently, significant associations between phubbing and the Dark Triad in Hispanic emerging adult college students have been shown [12].

Several instruments have been developed to measure phubbing [1,9,13]. For instance, the General Scale of Phubbing [GSP) and the General Scale of Being Phubbing (GSBP) [13] are two complementary scales that aim to obtain information about the perspective of the phubber, and the phubbee. Robert’s scale of partner phubbing (Phubbing) [9] focuses on measuring perceived phubbing. Finally, the Phubbing Scale (PS) is a ten-item scale that measures phubbing behavior along the two dimensions of phone obsession and communication disturbance.

Initially, the authors tested both one-factor and two-factor models, each including the original 10 items. However, these two models did not exhibit invariance across all countries. Subsequently, the authors refined the scale to consist of just eight items, henceforth referred to as the PS-8. They provided evidence supporting a two-factor structure (with four items per factor) that remained invariant across 17 countries, including Spain [14]. However, no other studies have analyzed the psychometric properties of shorter versions of the PS, including the PS-8 in its Spanish version.

In many research, clinical, and educational contexts, a brief version of the instrument is of considerable value for speed and ease of use. For example, this may facilitate screening for phubbing behaviors in school contexts where time is a key variable. Furthermore, evidence of validity is central to meaningful results across languages and cultures. In this context, we are keen to develop the psychometric adequacy of a shorter version of the PS in representative samples of the Spanish adolescent population. Therefore, the main objective of this research was to analyze the psychometry adequacy of a short form of the PS Spanish version [15] in the adolescent and young student population. The specific objectives were (a) to study the prevalence of phubbing in adolescent students; (b) to study the internal structure of a shorter version of the PS; (c) to gather evidence about the Measurement Invariance of a shorter version of the PS by gender and educational level; (d) to obtain evidence of the relationship between phubbing scores and socioemotional adjustment and mental-health indicators; and (e) to obtain evidence about the reliability of the scores of the shorter version of the PS scores.

## 2. Materials and Methods

### 2.1. Study Population and Data Collection

Participants were conveniently selected from educational institutions and universities. Each participant individually completed a questionnaire, which included a variety of scales. Informed consent was obtained for those participants under 18 years old. The time required to complete the survey was around 20 min, responding at their educational center with previous explanations about the questionnaire. This study was conducted with the approval of the Research Ethics Committee of the University of La Rioja (Code: inf_CE_46_2023).

### 2.2. Instruments

The Phubbing Scale (PS) [1] consists of 10 questions assessed on a Likert scale, ranging from 1 (never) to 5 (always). Phubbing behavior is evaluated through two main dimensions: Communication Disturbance (CD) (People complain about me dealing with my mobile phone) and Phone Obsession (PO) (I feel incomplete without my mobile phone) [14]. Despite showing adequate psychometric qualities, the research is still restricted by the lack of diverse sample populations [15,16]. The validated PS for the Spanish population has been used in this study [15].

The Strengths and Difficulties Questionnaire (SDQ) [17] is a self-report measure with 25 questions divided into five subscales, each containing five items: emotional problems (I worry a lot), behavioral problems (I get very angry and often lose my temper), peer problems (I get on better with adults than people of my age), hyperactivity (I am constantly fidgeting or squirming), and prosocial behavior (I am kind to younger children). The Spanish version of this scale, which has been translated and validated, has shown good psychometric properties in adolescent populations [18].

The Rosenberg Self-Esteem Scale (RSE) [19] is a widely recognized instrument for measuring self-esteem. It includes 10 items related to self-perception and self-assessment, with responses rated on a four-point scale based on the grade of agreement.

Higher scores reflect greater self-esteem. For this research, the Spanish version of the scale was used [20], and we found a McDonald’s Omega reliability coefficient of 0.798 for the total score.

The Interpersonal Emotion Regulation Questionnaire (IERQ) [21], consists of 20 items distributed across four factors: Enhancing Positive Affect (I like being around others when I’m excited to share my joy), Perspective Taking (Having people telling me not to worry can calm me down when I am anxious), Soothing (I look to others for comfort when I feel upset), and Social Modeling (It makes me feel better to learn how others dealt with their emotions). These factors represent the inclination to rely on others to boost positive emotions, gain new perspectives, find comfort through interpersonal interactions, and learn emotion regulation strategies by observing others. In this study, the Spanish version of the IERQ was used [22], with a McDonald’s Omega reliability coefficient of 0.918 for the total score.

The Compulsive Internet Use Scale (CIUS) [23] is a 14-item self-report measure aimed at assessing the severity of internet addiction or problematic internet use (PIU), including compulsive or pathological behaviors. Responses are rated on a 5-point Likert scale ranging from 0 (“never”) to 4 (“very often”), with the total score reflecting the level of PIU severity. In this study, the scale demonstrated strong internal consistency, with a McDonald’s Omega coefficient of 0.912.

The Personal Wellbeing Index—School Children (PWI-SC) [24] is a self-report instrument designed to evaluate subjective well-being and quality of life (QoL) in children and adolescents. It includes seven items that assess satisfaction across key life areas, such as standard of living, health, achievements, relationships, safety, community belonging, and future prospects. Each item is rated on a scale from 0 (“very sad”) to 10 (“very happy”). This study utilized the Spanish adaptation of the PWI-SC, which has demonstrated reliable psychometric properties [25].

In sum, the questionnaires used in this study included a variety of validated scales to examine the relationship between PS (see Appendix A) and various indicators of well-being and mental health challenges.

### 2.3. Data Analysis

We studied the descriptive statistics and the percentage distribution of the PS items. Then, we conducted a Confirmatory Factor Analysis (CFA) attending to the unidimensional model [14] of 8 items (items 1, 2, 3, 4, 6, 7, 8, and 9) in order to gather evidence about the internal structure of the PS. In addition, considering the lack of adequacy of this model, we tested a unidimensional model with six items and a two-dimensional model with six items. Item reduction was based on the study of factor loadings (lower than 0.30) and the discrimination indices (lower than 0.30). We used the Muthen’s quasi-likelihood estimator. The goodness-of-fit indices used were Chi-square (X2), Confirmatory Factor Index (CFI), Tucker Lewis Index (TLI), Root Mean Square Error of Approximation (RMSEA), and Standardized Root Mean Square Residual (SRMR). RMSEA values should be under 0.80 for a good model fit [26]. In addition, CFI and TLI over 0.95 are preferred. With regards to SRMR, values less than 0.08 can be considered as adequate. We also analyzed the MI by gender, educational level, and age. We conducted multigroup comparisons through structural equation modeling under the measurement models [27]. The change in CFI values (∆CFI) was a valuable criterion to determine if nested models were practically equivalent instead of ∆χ2 [28], as this one was more affected by changes in sample size. We then studied the internal consistency of the scores using McDonald’s Omega. We also gathered sources of valid evidence of the PS based on the correlations between the PS scores and different indicators of well-being and mental health by means of Pearson’s correlations. SPSS 24.0 (IBM, 2016) and JASP 0.18.3 (JASP, 2019) were used for data analyses.

## 3. Results

### 3.1. Characteristics of the Study Population

A total of 1351 Spanish adolescent students, aged between 12 and 21, participated in this study. Attending to gender, 733 were male (54.26%) and 578 female (42.74%), the rest of the participants preferred not to display their gender or indicated other. With regards to educational level, a total of n = 789 participants were enrolled in non-university studies (58.40%), including secondary education, high school, and vocational training, while the remaining participants n = 562 (41.60%) were pursuing university degree programs. Furthermore, a total of n = 679 of the participants were 12–17 years old (50.25%), and the rest n = 672 were 18–21 years old (49.75%).

### 3.2. Descriptive Statistics for the PS-6 and Prevalence Rates

Phubbing was prevalent among adolescent students. Specifically, 21.91% reported that they “almost always” kept their phone within reach (Item 6), while 20.65% indicated that they “always” did so. Additionally, 38.86% stated that they “always” checked their phone immediately after waking up in the morning (Item 7). In contrast, only 1.78% reported that they “always” receive complaints from others about their mobile phone use (Item 4), and just 0.81% selected the highest option (“always”) for Item 4 when referring to interactions with family.

Descriptive statistics for the six-item bidimensional model (see Table 1), show the prevalence of all the answer options for the SP-6, means, and standard deviations (SD).

### 3.3. Evidence of Validity Based on Internal Structure

Firstly, we analyzed the one-dimensional and the two-factor model for the 8-item model. As can be seen, goodness-of-fit indices did not reach the proposed cut-off indices (see Table 2). Then we analyzed a one-dimensional and a two-factor solution with six items after item reduction was conducted. The adequacy of the one-dimensional model was still questionable. In the case of the two-dimensional solution, all the goodness-of-fit indices were adequate. In addition, the study of factor loadings for this version revealed that all of them were statistically significant and over 0.30 (see Table 3).

### 3.4. Measurement Invariance of the PS-6 by Gender and Studies

Once we confirmed that the two-factor solution with six items was the most adequate, we proceeded to analyze the measurement equivalence of this structure across gender and educational level. To test this, we considered male and female participants for gender, non-university and university students for educational level, and adolescents and young adults for age. The ΔCFIs lower than 0.01 confirm strong MI by gender, educational level, and age (see Table 2).

### 3.5. Relation of the PS-6 with Different Indicators of Well-Being and Mental Health Problems

We studied the correlations between the PS-6 scores and variables related to socio-emotional adjustment (see Table 4). PS-6 scores were significantly correlated with indicators of well-being and mental health with the exception of the Hyperactivity and Peer Problems subscales of the SDQ. The PS dimensions and the PS Total score were positively correlated with the Total score of the SDQ and with difficulty’s subscales of the SDQ and with the total score of the CIUS, and negatively associated with indicators of psychological well-being (total score of the PWI-SC) and self-esteem (total score of the Rosenberg). Moreover, the PS-6 scores were very strongly associated with the PS original form (r = 0.927).

### 3.6. Study of the Reliability of the Scores of the PS-6

Finally, we calculated the McDonald’s Omega coefficient as an index of internal consistency of the scales. The total score displayed a coefficient of 0.701, and the Communication and Obsession dimensions revealed coefficients of 0.617 and 0.669, respectively. All the discrimination indices were over 0.30.

## 4. Discussion

Smartphone access and use among adolescents and young adults is becoming, in European countries, almost ubiquitous [29]. Time spent on electronic devices such as smartphones has increased during and since the world pandemic COVID-19, not only for adults but also for adolescents. Consequently, Phubbing is receiving more attention and research with the aim to better understand and screen for a behavior that has been linked with psychological difficulties like, among others, poorer communication quality [5,6], diminished interpersonal interactions [7,8], and tendencies towards addictive behaviors [15]. Therefore, screening at early stages seems relevant, and, consequently, assessment tools with adequate evidence of validity and reliability are highly necessary. Thus, the main goal of the present study was to analyze the psychometric adequacy of a short form of the PS in its Spanish version.

According to our results, phubbing rates were high among adolescents and young adults. The mean for the Communication Disturbance and Phone Obsession subscales were 6.52 and 9.31. Moreover, 21.91% indicated the option “almost always” when asked if they kept their phone within reach, while 20.65% revealed that they “always” did so. Furthermore, 38.86% of the total sample selected the option “always” when asked about whether they checked their phone immediately after waking up in the morning (Item 7). These results seem to be in line with previous research [30,31,32,33] that revealed that phubbing is a prevalent phenomenon among adolescents and young adults.

The results found in the present study about the internal structure of the PS affirm that the PS-6 two-dimensional model showed adequate goodness-of-fit indices. A study across 20 countries, including Spain, indicated that a shorter form of the PS with 8 items better fit the data than the original 10-item version [18]. Similarly, a study conducted in Spain revealed that the PS 10-item version had adequate evidence of internal structure but only after adding some correlated errors, also indicating that the 8-item version was not adequate [34].

After the PS-6 two-factor structure was confirmed as the most satisfactory solution, we, further confirmed that the dimensional structure could be replicated with measurement invariance across gender, educational level, and age. Previous studies, similarly to our results, revealed MI of the PS in the original version [35,36] and in the 8-item shorter version [14,37]. Nonetheless, information about the measurement equivalence of the instrument in shorter forms across variables like gender, age, or educational stages is not available. This information is critical to be able to compare male and female scores or samples, high school and university students, and adolescents and young adults for example.

In addition, we also found consistent evidence of the relationship of the PS-6 with different indicators of mental health and psychological well-being. Thus, higher scores on phubbing were related to higher levels of psychological difficulties and problematic internet use. Also, phubbing was associated with lower levels of psychological well-being and self-esteem. This is consistent with previous studies [6,38,39,40,41,42,43]. Overall, the PS-6 showed acceptable reliability, consistent evidence of construct validity, and almost perfectly linear relationships with the original PS. When analyzing the relationship between Phubbing and other indicators of psychological well-being like hyperactivity and peer problems, no relationship was found. A possible explanation for this could be the fact that the hyperactivity subscale refers to both inattention and hyperactive symptoms. It could be the case that if we focus only on the attentional deficit, a positive correlation would be found. Also, as the PS refers to phubbing behaviors, more information should be gathered about the impact of these behaviors not only on the person but also on others to further understand the relationship between phubbing and potential peer problems. It is worth noting that no previous studies have analyzed the relationship of phubbing with these indicators, so future research could explore this in order to develop evidence-based educational intervention programs and wellness plans to mitigate the potential negative effects of this phenomenon.

There are several limitations such as the use of self-report measures, especially in adolescent populations. Therefore, future research should consider the inclusion of objective measures of phubbing behavior, which would provide valuable support to the subjective data obtained through participants’ self-reports and offer a more comprehensive picture of phubbing habits. Moreover, the cross-sectional nature of the study limits the establishment of cause-effect relationships. Thus, longitudinal studies could extend our understanding of the causes and consequences of phubbing among adolescents. This would contribute valuable insights into the causal relationship between phubbing and other psychological problems, including socio-emotional issues. Finally, we studied adolescent and university students from a specific region using convenience sampling, so the results found may not be equivalent to other cultural contexts or entirely representative. Therefore, future studies on the psychometric properties of the PS in different areas are necessary.

## 5. Conclusions

Notwithstanding these limitations, the present study work contributes valuable information to understanding phubbing among adolescent and young adult populations. In addition, and to the best of our knowledge, this is the first study providing evidence of the psychometric adequacy of a short form of the PS in its Spanish version and in adolescent and young adult populations. To sum up, the PS-6 is a brief and easy-to-use screening instrument that allows one to study Phubbing, a phenomenon that is receiving more attention due to its implications for mental health and psychological well-being.

## Figures and Tables

**Table 1:** Prevalence rates for the 6-item Phubbing Scale (PS-6) and descriptive statistics for the subscales and the different items in the total sample.

	Prevalence					Descriptive Statistics			
Items	Never	Rarely	Sometimes	Almost Always	Always	Mean	SD	Skewness *	Kurtosis **
Communication Disturbance						6.52	2.03	0.76	0.84
1	7.30	40.10	35.80	13.80	2.90	2.65	0.91	0.42	−0.03
3	48.93	33.16	11.62	4.52	1.78	1.77	0.95	1.3	1.38
4	24.43	47.45	22.06	4.81	0.81	2.1	0.85	0.6	0.28
Phone Obsession						9.31	2.69	0.02	-0.53
6	17.91	19.91	19.62	21.91	20.65	3.07	1.4	−0.07	−1.28
7	31.90	33.68	20.36	10.07	4.00	2.21	1.12	0.72	−0.25
8	29.61	43.75	20.65	4.44	1.48	2.04	0.9	0.77	0.5
Total Score						15.83	4.04	0.30	0.07

* Skewness SE 0.067. ** Kurtosis SE 0.133.

**Table 2:** Goodness-of-fit indices for the hypothetical model tested and measurement invariance across gender and age for the PS-6 two-dimensional model.

Model	χ^2^	*df*	CFI	TLI	RMSEA (IC 90%)	SRMR	ΔCFI
8 items 1 factor	987.258	9	0.803	0.821	0.123 (0.116–0.131)	0.094	
8 items 2 factor	589.687	8	0.86	0.854	0.120 (0.112–0.127)	0.072	
6 items 1 factor	173.927	9	0.935	0.891	0.118 (0.103–0.134)	0.060	
6 items 2 factor	65.223	8	0.977	0.957	0.073 (0.058–0.091)	0.039	
Measurement Invariance							
Gender							
Male (n = 733)	48.970	5	0.975	0.954	0.068 (0.061–0.075)	0.033	
Female (n = 578)	47.872	5	0.974	0.969	0.069 (0.062–0.075)	0.029	
Configural invariance	69.977	16	0.979	0.960	0.0072 (0.055-0.089)	0.040	
Metric invariance	66.569	20	0.982	0.972	0.060 (0.044-0.076)	0.043	−0.01
Scalar invariance	96.611	36	0.976	0.98	0.051 (0.039–0.063)	0.042	−0.01
Educational Level							
Non-University (n = 789)	48.264	5	0.977	0.955	0.066 (0.043–0.072)	0.032	
University (n = 562)	47.823.	5	0.976	0.951	0.063 (0.045–0.080)	0.026	
Configural invariance	46.391	16	0.977	0.954	0.064 (0.046–0.083)	0.027	
Metric invariance	49.585	20	0.978	0.968	0.053 (0.038–0.070)	0.030	−0.01
Scalar invariance	51.787	36	0.966	0.962	0.059 (0.045–0.073)	0.032	−0.01
Age							
12–17 (n = 679)	48.365	5	0.974	0.955	0.069 (0.047–0.073)	0.032	
18–21 (n = 672)	48.821	5	0.969	0.951	0.061 (0.044–0.079)	0.025	
Configural invariance	47.290	16	0.972	0.954	0.064 (0.040–0.079)	0.028	
Metric invariance	49.394	20	0.968	0.968	0.052 (0.035–0.068)	0.029	−0.01
Scalar invariance	50.986	36	0.966	0.962	0.057 (0.044–0.071)	0.031	−0.01

**Table 3:** Factor loadings for the PS-6 two-dimensional model.

	Factor Loadings	SE	CI 95%	
Item			Lower	Upper
Factor 1				
1	0.805	0.024	0.758	0.852
3	0.438	0.029	0.381	0.495
4	0.652	0.024	0.606	0.698
Factor 2				
6	0.600	0.025	0.551	0.649
7	0.796	0.023	0.651	0.740
8	0.695	0.023	0.649	0.741

**Table 4:** Pearson’s Correlations between the PS-6 two-dimensional model and different indicators of well-being and mental health problems.

Variable	PS Communication	PS Obsession	PS-6 Total
PS Obsession	0.452 **	—	
PS-6 Total	0.802 **	0.895 **	—
Rosenberg TOTAL	−0.145 **	−0.130 **	−0.159 **
CIUS TOTAL	0.417 **	0.363 **	0.450 **
SDQ EMO	0.159 **	0.167 **	0.189 **
SDQ CON	0.160 **	0.039	0.109 **
SDQ PEER	0.009	0.027	0.025
SDQ HYPER	−0.009	−0.045	−0.034
SDQ PROS	0.081 *	−0.044	0.010
SDQ TOTAL	0.162	0.104 **	0.152 **
PWI TOTAL	−0.080 *	−0.115 **	−0.115 **
IERQ TOTAL	0.099 **	0.105 **	0.120 **
PS-10 Total	0.817 **	0.795 **	0.940 **

** The correlation is significant at 0.01 level (bilateral). * The correlation is significant at 0.05 level (bilateral). PS-6 Total = Total score of the Phubbing Scale 6 items; Ps Communication = Phubbing Scale Communication; PS Obsession = Phubbing Scale Obsession; SDQ EMO = SDQ Emotional Problems; SDQ CON = SDQ Conduct Problems; SDQ PEER = SDQ Peer Problems; SDQ HYPER = SDQ Hyperactivity; SDQ PROS = SDQ Prosocial; PS-10 Total = Total score of the Phubbing Scale 10 items; IERQ TOTAL = Internal Emotion Regulation Questionnaire Total Score; PWI TOTAL = Personal Well-being Index-School Children.

## Data Availability

The data are not available as they contain sensitive information about human subjects and consent was not obtained for its dissemination.

## Appendix A

Items of the Spanish Phubbing Scale:

Alteración en la comunicación

1. Estoy pendiente de mi teléfono móvil cuando estoy en compañía de otras personas
2. Estoy ocupado con mi teléfono móvil cuando estoy con mis amigos
3. Otras personas se quejan sobre mi uso del teléfono móvil
4. Estoy ocupado con mi móvil cuando estoy con mis familiares
5. A mi pareja le molesta que esté ocupado con el móvil (o familiares si no tienes pareja)

Obsesión por el teléfono

6. Mi teléfono móvil está a mi alcance
7. Lo primero que hago al despertarme es mirar mis mensajes en el móvil
8. Me siento vacío sin mi móvil
9. Cada día aumenta mi uso del teléfono móvil
10. El tiempo que dedico a actividades sociales, personales o profesionales, se reduce por el tiempo que uso el móvil
